# Is Calorie Labeling on Menus Related to Weight Disturbances among Females in Saudi Arabia?

**DOI:** 10.1155/2021/4041451

**Published:** 2021-09-03

**Authors:** Hala Al-Otaibi, Tahani Al-Sandal, Haiam O. Elkatr

**Affiliations:** ^1^Department of Food and Nutrition Science, College of Agricultural Science and Food, King Faisal University, Hofuf, Saudi Arabia; ^2^Department of Home Economics, Faculty of Specific Education, Ain Shams University, Cairo, Egypt

## Abstract

Calorie labeling is a recent initiative from the Saudi Food and Drug Authority (SFDA) aimed to reduce the prevalence of noncommunicable diseases (NCDs) by influencing people to make healthier food choices when they eat out and can also help people with weight disturbances to be more aware of their calorie intake. The present study aimed to investigate the association between the use of calorie labeling on restaurant menus, calorie intake, weight concern, body weight perception, and weight-control behaviors among young women. A quasi-experimental study was conducted among female students at a university restaurant. Participants were assigned to two groups: food menus with (experimental group) and without (control group) calorie labeling. The logistic regression model assessed the predictors of using calorie information separately for the experimental and control groups. Calorie labeling had a significant effect on reducing calorie consumption in the experimental group by 59 calories compared to the control group. The higher weight concern in the control group (OR = 0.410; 95% CI 0.230–0.730; *P* ≤ 0.002) was a predictor for using calorie information. The experimental group had higher weight concern (OR = 1.530; 95% CI 1.107–2.115; *P* ≤ 0.01) and body weight perception (OR = 4.230; 95% CI 1.084–6.517; *P* ≤ 0.038) and lower calorie intake (OR = 1.005; 95% CI 1.001–6.517; *P* ≤ 0.008) predictors for using calorie information. Weight-control behaviors did not significantly predict the use of calorie information in the groups. Calorie labeling might increase the weight disturbances among young females. More investigation is needed across various populations to gain a better understanding of calorie labeling as an effective food choice among people who are vulnerable to weight disturbances or already have weight disorders.

## 1. Introduction

Obesity is an excessive accumulation of fat inside the body's tissues, which is harmful to a person's health. It has been classified as a chronic disease and a major public health problem [1] as it increases people's susceptibility to many chronic diseases such as cancers and cardiovascular diseases. These diseases account for approximately 71% of deaths worldwide [2]. Globally, studies have shown that obesity rates have risen dramatically, nearly tripling between 1975 and 2016. In 2016, more than 1.9 billion adults were overweight and more than 650 million people were obese [2]. If no action is taken to counter the spread of obesity, it is estimated that approximately half of the world's population will be overweight or obese by 2030 [3].

Saudi Arabia has witnessed significant cultural development over the last few decades, which has led to a difference in lifestyle, an increase in the prevalence of obesity to 33.7%, and an increase in the proportion of overweight inhabitants by 68.2% [4]. Thus, obesity has become a major public health concern, and seven of ten people in Saudi Arabia are either obese or overweight [5]. This is attributed to the spread of sit-down restaurants, fast-food restaurants, coffee shops, and home delivery services, which contribute to a higher calorie intake than the daily requirement.

In recent years, many government initiatives have emerged that aim to raise public health awareness among individuals and communities. In addition, they addressed the quality of food required to help reduce the prevalence of obesity among citizens and maintain healthy lifestyles. One of the most recent programs that align with the strategic objectives of Saudi Arabia's 2030 vision is the Saudi Food and Drug Authority (SFDA) initiative to reduce noncommunicable diseases (NCDs). The initiative was implemented in January 2019, and all restaurants and coffee shops had to comply with its requirements. It states that all out-of-home foodservice providers must clearly list all the calories on monitors and printed menus using “calories” or “kilocalories,” informing consumers about the calorie content of meals, promoting healthier eating in the process [6, 7].

Worldwide, providing calorie labeling is one of the most prominent policy interventions to reduce the prevalence of NCDs like obesity. Exposure to calorie information over time through restaurant menus might increase consumer awareness of calories, encourage healthy food choices, help to reduce calorie consumption, change people's attitudes, and make them pay more attention when ordering food from restaurants [8, 9]. Recent studies in the Saudi population have assessed the knowledge, attitudes, and practice of utilizing caloric information in restaurant menus and found that 24–50% utilized the caloric information on menus when purchasing meals from restaurants [10–12].

Usually, calorie labeling targeted obese and overweight individuals to reduce excessive caloric intake; however, sometimes, the nontargeted individuals (eating and/or weight disturbances) may be using this caloric information in a negative way [13]. On the other hand, research suggests that some individuals who use calorie information when dining out have unhealthy weight-control behavior and have more weight concerns than those who do not utilize calorie information [13, 14].

Currently, few studies have investigated the effects of calorie labeling on individuals with eating and/or weight disturbances [13–16]; based on their findings, there is a gap in the literature on calorie labeling, food choice, and eating and/or weight disturbances which are still not well characterized in the literature. In Saudi Arabia, calorie labeling is a new policy that has been published to study the changes in Saudi customers' behavior, as calorie labeling influences their calorie intake, knowledge, and attitude [10–12]. To date, no study in Saudi Arabia has investigated the influence of calorie labeling on individuals with eating and/or weight disturbances. To study this gap in the literature, we aimed to employ a quasi-experimental design to investigate the association between the use of calorie labeling on restaurant menus, calorie intake, weight concern, body weight perception, and weight-control behaviors among young females. However, previous studies found that young females are more likely to use calorie labeling [10–12] and usually have weight concerns with misperceptions about weight status [15, 16]. Additionally, the current study determines whether more searches in this area are needed.

## 2. Methods

### 2.1. Participants

**Figure 1:**
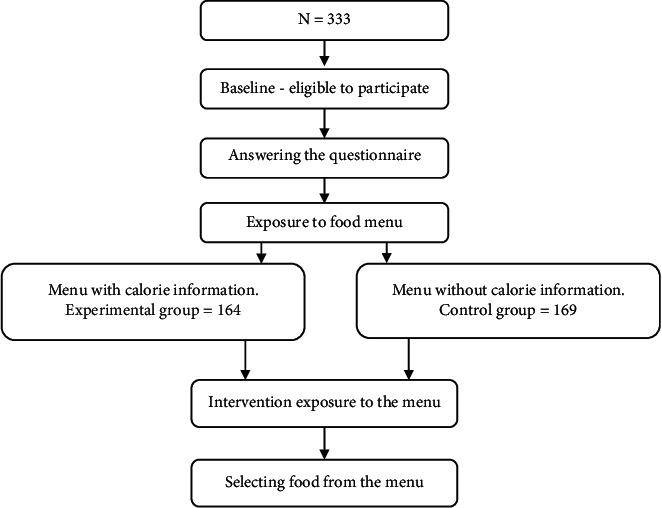
Study design.

The sample consisted of 333 undergraduate female students at King Faisal University, located in Al-Ahsa City, Saudi Arabia. Participants were recruited from a female campus representing all the colleges (Figure 1).

### 2.2. Procedure

**Figure 2:**
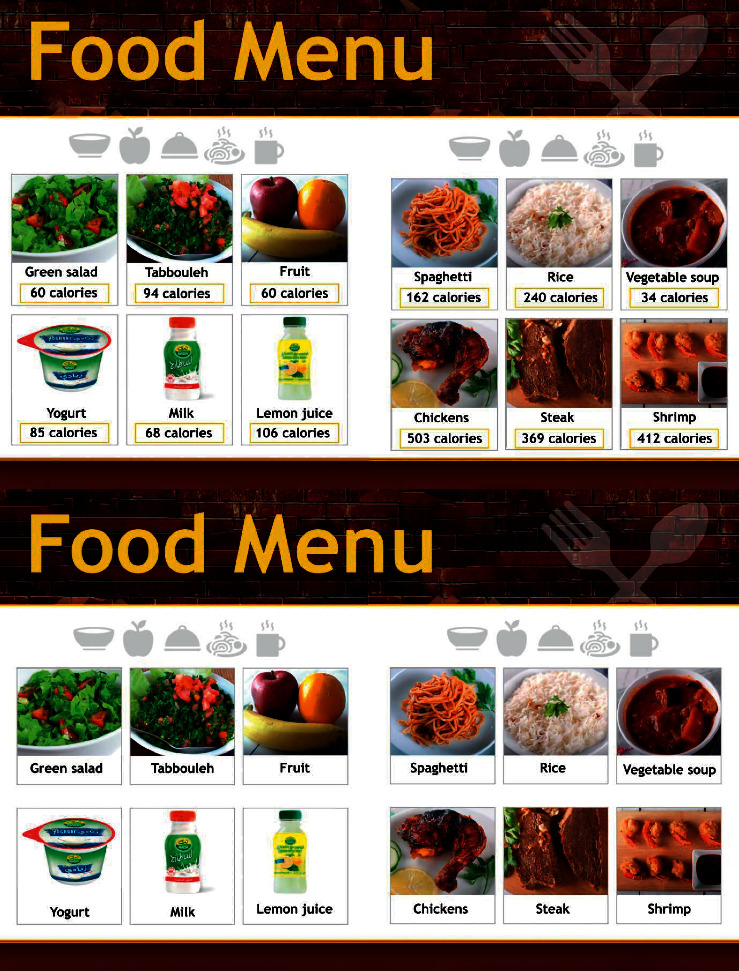
Menus without and with calorie information.

A study with a quasi-experimental design was conducted on female university students. The researchers met with the participants at the university restaurant (lunchtime 11 AM–2:30 PM), and once informed consent was obtained, we asked them to complete the questionnaire, and the researcher took their anthropometric measurements through short interviews (approximately 10 minutes). Participants who answer “strongly agree” in weight concern section for more than one question were assigned to the experimental group with a calorie-labeled menu, and the rest of participants were assigned to the control group without a calorie-labeled menu (Figure 1). Participants were asked to select food items from the menu that they would like to eat for lunch (Figure 2). Participants who were pregnant, had chronic diseases, or were less than 18 years old were excluded. The control group (non-calorie-listed menu) comprised 169 participants, representing 50.8% of the sample size. The experimental group (calorie-listed menu) comprised 164 participants, representing 49.2% of the study sample. This study was approved by the Research Ethics Committee of the King Faisal University.

### 2.3. Study Variables

- Social and economic information: this section contained three questions about age, marital status, and monthly household income.
- Body measurements: measurements were taken from all participants. Height was measured using a meter (Seca), with participants barefooted. Weight was measured in kilograms using an electronic digital scale (OMRAN HN286). Weight and height are required to determine the body mass index (BMI), which was calculated using the formula weight (kg)/height (m^2^) [17].
- Calorie information on restaurant menus: participants were asked if they noticed any calorie information on the restaurant menus and if that information affected their item selection. In addition, participants were asked questions to determine the effect of listed calorie information on their food selection. They could answer these questions by selecting one of the four options, including ordering a smaller meal (Cronbach's alpha = 0.72) [8, 13]. Participants were also asked about the daily calorie recommendations for women aged 18 years or more.
- Body weight perception: to measure participants' perceptions of their weight, they were asked one question about their perception of their weight (how do you describe your body weight?). They could answer the question by selecting one of the four options, including thin, normal, overweight, and obese. Thereafter, their answers were compared with their actual BMI and categories such as underperception (their perceived weight is less than the actual weight), accurate perception (their perceived weight is equal to the actual weight), and overperception (perceived weight is more than the actual weight) [15, 18].
- Weight concern: participants were assessed using three statements related to their weight, such as “I am worried about gaining weight.” Participants' agreement with these statements was determined by their selection of the following options: strongly disagree = 1, disagree = 2, agree = 3, and strongly agree = 4. The higher the value, the greater the concern. The value of Cronbach's alpha was 0.84, with a possible score between 3 and 12 [13].
- Weight-control behaviors: this section had one question about participants who lost weight or prevented themselves from gaining weight during the previous year. They could answer these questions by selecting either Yes or No. This included four healthy methods, ate less sweets and high-fat food, did exercise, watched my portion sizes, and ate less calories, and five unhealthy methods, made myself vomit, fasted for long hours, took diet pills, did smoking, and used a food substitute (powder/special drink). For analysis, participants who answered Yes for one or more healthy behaviors were classified as having healthy weight control and participants who answered Yes for one or more unhealthy behaviors were classified as having unhealthy weight control [13, 19].
- Menu: this included two menus, consisting of 12 food items each, offered by the university restaurant in the female campus for lunch. The two identical menus were designed in the same way in terms of shape, size, and format. However, the first menu contained only food items without calorie information, while the second contained food items with calorie information (Figure 2). No prices were included in the menus because of their potential to influence the participants' selection.

### 2.4. Data Analysis

The collected data were analyzed using SPSS (version 23.0). Statistical analysis was conducted using an independent *t*-test and analysis of variance (for the differences between the means). The chi-square test was used to assess the independence of categorical variables. The logistic regression model assessed the predictors of using calorie information (no reference category) separately for the experimental and control groups.

## 3. Results

The mean age of the participants in both groups was 20 years. The majority of participants were unmarried, and more than 70% of groups had monthly family household incomes exceeding 5000 riyals.  Table 1 shows a decrease in calorie intake in the experimental group compared to the control group by 59 calories, with a significant difference between the two groups. The mean BMI for the two groups was similar, without any significant difference. Significantly more participants in the experimental group (83.5%) noticed the calorie information on the restaurant menus and were influenced by the calories when placing their orders (64.6%) than in the control group (*P* < 0.001). However, two-thirds of the experimental group ordered a smaller portion size, and 40.3% of the control group ordered their regular meals without any change, with the difference between the above-listed percentages being statistically significant (*P* < 0.001). The results show that more than half of the participants in both groups knew that women needed approximately 2,000 calories per day.

The majority of the participants in the control group had accurate body weight perception (61.1%), and 42.1% of the participants in the experimental group had a significant difference in body weight perception (*P* < 0.001). The mean weight concern in the experimental group was higher than that in the control group, with a significant difference between the groups and the three statements. Meanwhile, 32.3% of the experimental group and 25% of the control group used unhealthy weight-control behaviors to lose weight or to prevent weight gain; however, there was no statistically significant difference between these percentages. More than half of the participants in the control and experimental groups (59.5% and 52.5%, respectively) had been fasting for long hours, an unhealthy behavior, and 25% of the participants in the experimental group vomited. The differences in these percentages between the two groups were statistically significant (*P* ≤ 0.042), as shown in Table 2.

According to body weight perception, the control group showed no significant difference among the three perceptions of calorie intake, but for the body weight concern, they had a significant difference (8.71 ± 2.76) (*P* < 0.009). In the experimental group, the participants with over-body weight perception had lower mean calorie intake (479.15 ± 203.79) and higher mean weight concern (9.27 ± 2.95) compared to under-/accurate body weight perception with a significant difference (*P* ≤ 0.029 and 0.036, respectively), as shown in Table 3.

Higher weight concern in the control group (OR = 0.410; 95% CI 0.230–0.730; *P* ≤ 0.002) is a predictor for using calorie information in the control group. The experimental group had higher weight concern (OR = 1.530; 95% CI 1.107–2.115; *P* ≤ 0.01) and body weight perception (OR = 4.230; 95% CI 1.084–6.517; *P* ≤ 0.038) and lower calorie intake (OR = 1.005; 95% CI 1.001–6.517; *P* ≤ 0.008) predictors for using calorie information. Weight-control behaviors did not significantly predict the use of calorie information in the groups.

## 4. Discussion

The current study described the use of calorie information in university restaurant menus among young females and investigated the association between weight concerns, body weight perception, and weight-control behaviors and using this information to limit calorie intake. Similar to previous studies, we found that listing calories on menus had an obvious effect on reduction of the experimental group's mean calorie intake by 59 calories, compared to the control group. This might be attributed to the calorie content of the items provided for the participants in the experimental group. This has led to the selection of lower calorie items or the selection of fewer items, consistent with the results of Krešić et al. [20]. Zlatevska et al. [21] found that listing calories on menus limited the consumption to 27–67 calories per meal.

When the participants were asked “Have you noticed the calories listed on restaurant menus?” question, the percentage of those who noticed them was high in both groups, which is in line with the findings of Rahamat et al. [8, 9] and local studies [10–12]. Furthermore, listing calories on menus is a new regulation in the community that attracts attention. This might have a link to or be a cause for the effect of lower calorie intake, with items lower in calories being ordered more by participants in the experimental group than those in the control group.

Although calorie information was high in the experimental group, most participants in the control group did not use the calories listed, consistent with the findings of Olivera et al. [22], who found that most participants did not use calorie information during the selection of food items and they did not know how to use or read calorie information. Moreover, some of them believed that calorie information was difficult to interpret and this was for nutritionists only. Other participants believed that focusing on calories would prevent them from enjoying their food.

The findings show that two-thirds of the participants in the control group did not utilize the calorie information when selecting food from the menu. They selected items that they were familiar with or that they wanted to eat without taking calories into account. However, 19% of the participants in the experimental group would sometimes replace a high-calorie meal with a meal containing fewer calories, and 60.2% ordered a smaller portion. This is consistent with the findings of previous studies [8, 23]. Seyedhamzeh et al. [24] found that there were many different factors influencing the selection of food items, such as taste, price, culture, and food awareness. A study by Robertson and Lunn [25] found that the priority factors that affected consumers when selecting a meal were taste (43%), followed by nutritional value (20%), hunger (19%), price (10%), and calorie content (8%), which might have caused 63.3% of the participants of the control group not to utilize calorie information when selecting food. Another study by Avcibasioglu et al. [26] revealed that although a high percentage of students utilized calorie information when ordering a meal, their choices were highly affected by factors other than calorie information. Price was the first factor affecting their choice (78%), followed by meal ingredients and meal size. Calories came in the fourth place, as only one-third of the students from the sample (30%) were influenced by them when choosing their meals, which is similar to what we observed in the control group.

One of the reasons why some of the participants were not noticed or affected by calorie information may be reading to the location of the calorie information on the menu; it is usually set out to the far left (Arabic menu) of the item's name. This leads to a lack of interest in reading the calories. In the American college campus study, calories were listed on the same location as the item's name and this reduced calorie intake by 16.31%. This means that the location of calorie information on the menu affects consumers' decisions [27]. This is considered in the present study. We design the menu without price, and we put the calorie information under the food item.

We found that most of the participants were aware of their average daily calorie needs. Approximately two-thirds of the participants in the control group and more than half of the participants in the experimental group chose the correct answer, which was 2,000 calories. This could be due to the fact that the average number of calories needed by a person daily is 2,000 and that the SFDA has recently made it mandatory for food providers to write it on all food menus. However, approximately one-third of the participants from both groups selected lower calories (1,800 calories), which was contrary to what was found in a study by Krešić et al. [20], conducted at the University of Croatia, where 53.7% of the participants in the experimental group and 44.8% of the participants in the control group correctly answered the question. Moreover, more than one-third of the Croatian students in both groups overestimated their daily calorie needs, which might be due to the fact that the people in the study sample had less awareness of the calories to be consumed daily and were not affected by the amount of calories written on the menus because they did not know how to use calorie information.

The majority of the participants in the control group had accurate body weight perception (62.1%) and low mean weight concern, and only 25% had unhealthy weight control. Fifty percent of the participants in the experimental group had accurate body weight perception, although 42.1% believed that they were overweight or obese with a higher weight concern (9.27 ± 2.95) and lower caloric intake (479.15 ± 203.79), which is in line with the findings of Larson et al. [13]. Reale and Flint [28] found that participants in the calorie information group reported significantly more weight concerns (3.1 ± 0.92) when they ordered meals than those in the control group (2.1 ± 1.08).

Consistent with our results, Kim et al. [18] found that women with overperception of their weight, aged 19–40 years, were associated with averagely more depressive symptoms and unhealthy weight control. Contrary to the present study, Lillico et al. [14] found that 80% of the participants had accurate body weight perception before and after adding calories to the menus in a pre-post intervention study. This difference might have been caused by their low mean weight concern compared to the present study, especially in the experimental group where 64.6% of the participants were influenced by the calorie information, which was in line with the conclusion of the study by Avcibasioglu et al. [26].

The current literature includes a limited number of studies examining the association between calorie labeling and eating and/or weight disturbances with mixed findings [13–16, 29]. Larson et al. [13] also found a significant association between weight concerns among experimental and control groups and the use of calorie information. Alternatively, Lillico et al. [14] found that calorie labeling did not significantly affect those at high risk for eating/weight disturbance among the participants of their study. Generally, positive weight perception is a basic weight-control requirement that reduces weight concern. This can help raise behavioral intention to make healthy weight-control decisions, such as selecting low-calorie foods, engaging in more physical activity, paying attention to program messages and initiatives, or treating subjects with eating/weight disturbances [30]. Overperception of body weight and lower calorie intake were significantly associated with the use of calorie information in the experimental group. A cross-sectional study conducted among 493 college students aimed to measure the associations between calorie-tracking devices and eating/weight disturbances found a significant association for lower calorie intake but not for weight concern and body weight perception, which might be due to the participants monitoring their calorie intake for reasons not related to eating or weight disturbances [29]. Contrary to the findings of the present study, Nianogo et al. [15] found that body weight perception was not significantly associated with using calorie information among participants with body weight perception but for participants with underperception of their body weight who selected lower calorie foods when using the calorie post on menus.

The present study had some limitations. First, the participants were not representative of the general population, as the sample of university students (females only) was small. The quasi-experimental design that lacks random selection was another limitation; however, to overcome this bias, we include a control group [31]. Second, we did not write the statement (adults need an average of 2,000 calories on a daily basis, and individual calorie needs may vary from person to person) on the calorie-listed menu, although this statement is required by the SFDA initiative. Third, the study was restricted to university restaurants and lunch items only (one location), and we are not investigating the other menus in the university cafeterias because of the variety of menu items that we would have ended up with, such as sandwiches and pastries. Fourth, in spite of weight disturbances being multidimensional, we used only three scales, which might have resulted in misclassification; therefore, more investigation is required. Finally, the eating disturbances not examined in the present study may have affected item selection and calorie intake.

In spite of these limitations, the present study has several strengths. First, the data and anthropometric measurements were collected through personal interviews, which helped to establish credibility and ensure that the responses were as accurate as possible. This also reduces the errors that may arise from female students not accurately mentioning their weight and height. Second, the absence of prices on the menu items meant that prices did not affect their meal selection. Third, the study was carried out in a real environment of a university restaurant. Fourth, the study menu provides a drink option that is usually offered in university restaurants to limit any alteration in the caloric intake of participants. Finally, the study contributes to supporting the SFDA's strategy to list calories and draw the community's attention to its messages.

## 5. Conclusions

To the best of our knowledge, the present study is the first to investigate the association between weight concerns, body weight perception, weight-control behaviors, and calorie information to limit calorie intake in Saudi Arabia. Calorie labeling had a significant effect on reducing calorie consumption among the participants of the experimental group, with a drop of 59 calories per meal compared to the control group. According to Guth [32], a reduction of 50 calories per day would result in a significant decrease in calorie intake per year, which would be equal to losing 2.27 kilograms. This represents approximately 3% of the average weight of a person. The study demonstrated that using calorie information is associated with more weight concerns (both groups), body weight perception, and calorie intake limit among young females in the experimental group. Weight-control behaviors were not significantly associated with the use of calorie information despite the fact that 25% of the participants in the control group and 32.3% of the participants in the experimental group had unhealthy weight-control behaviors, where 53 participants fasted for long hours and 20 participants vomited. Calorie labeling might increase weight disturbances among young females with some symptoms of weight disturbance. More investigation is needed to assess whether calorie labeling is an effective food choice measure among people who are predisposed to eating/weight disturbances or already have eating/weight disorders in various populations.

## Figures and Tables

**Table 1:** Calories, anthropometric measurements, calorie information, weight concern, weight perception, and weight-control behaviors in the two groups (*N* = 333).

Variables	Control group, 169 (50.8%)	Experimental group, 164 (49.2%)	*P*

Age/years (mean ± SD)	20.46 ± 1.83	20.30 ± 1.71	0.388^$^
Marital status			
Unmarried	134 (81.7%)	137 (81.1%)	0.880^#^
Married	30 (18.3%)	32 (18.9%)	
Monthly income (SR)			
Less than 5000	49 (29.8%)	47 (27.8%)	0.706^#^
More than 5000	115 (70.2%)	122 (72.2.1%)	
Calories (mean ± SD)	585.20 ± 233.41	526.10 ± 205.02	0.015^*∗*^^$^
Weight (kg) (mean ± SD)	54.40 ± 11.10	54.02 ± 10.37	0.752^$^
BMI kg/(m^2^) (mean ± SD)	22.04 ± 4.12	21.93 ± 4.14	0.815^$^
BMI categories			
Underweight	33 (19.5%)	36 (22%)	0.882^#^
Normal	99 (58.6%)	89 (54.3%)	
Overweight	29 (17.2%)	30 (18.3%)	
Obese	8 (4.7%)	9 (5.4%)	
Have you noticed any calorie information while purchasing a meal or snack in any type of restaurant?			
Yes	134 (79.3%)	137 (83.5%)	0.001^*∗∗*^^#^
No	35 (20.7%)	27 (16.5%)	
Did you use that calorie information when deciding what to order?			
Yes	62 (36.7%)	106 (64.6%)	0.001^*∗∗*^^#^
No	107 (63.3%)	58 (35.4%)	
How did you use that calorie information when deciding what to order? *Only for who answer the previous question yes.*			
(1) Avoided ordering high-calorie menu items.	4 (6.5%)	3 (2.8%)	0.001^*∗∗*^^#^
(2) Sometimes I do not order high-calorie food.	15 (24.2%)	20 (19%)	
(3) Decided on a smaller portion size.	18 (29%)	64 (60.2%)	
(4) I ordered my regular meal. Calories do not affect me.	25 (40.3%)	19 (18%)	
Women's daily calorie needs are			
(1) 1,800 calories	49 (29.0%)	56 (34.2%)	0.597^#^
(2) 2,000 calories	106 (62.7%)	95 (57.9%)	
(3) 2,500 calories	14 (8.3%)	13 (7.9%)	

#Chi-square test, $*t*-test, ^*∗∗*^*P* ≤ 0.001, and ^*∗*^*P* ≤ 0.05, SR: Saudi riyal.

**Table 2:** Body weight perception, weight concern, and weight-control behaviors in the groups (*N* = 333).

Variables	Control group, 169 (50.8%)	Experimental group, 164 (49.2%)	*P*

Body weight perception			
Underperception	29 (17.2%)	13 (7.9%)	0.001^*∗∗*^^#^
Accurate perception	105 (62.1%)	82 (50%)	
Overperception	35 (20.7%)	69 (42.1%)	
Weight concern			
(1) I think a lot about being thinner.			
Strongly disagree	37 (43.2%)	53 (32.3%)	0.026^*∗*^^#^
Disagree	6 (3.6%)	18 (11%)	
Agree	35 (20.7%)	38 (23.2%)	
Strongly agree	55 (32.5%)	55 (33.5%)	
(2) I am worried about gaining weight.			
Strongly disagree	65 (38.5%)	39 (23.8%)	0.013^*∗*^^#^
Disagree	7 (4.1%)	14 (8.5%)	
Agree	30 (17.8%)	27 (16.5%)	
Strongly agree	67 (39.6%)	84 (51.2%)	
(3) I sometimes skip meals since I am concerned about my weight.			
Strongly disagree	106 (62.2%)	52 (31%)	0.001^*∗∗*^^#^
Disagree	14 (8.3%)	34 (20.7%)	
Agree	41 (24.3%)	36 (22%)	
Strongly agree	8 (4.7%)	42 (25.6%)	
Total weight concern (mean ± SD)	6.72 ± 2.98	7.94 ± 3.13	0.001^*∗∗*^^$^
Weight-control behaviors			
Healthy	127 (75%)	111 (67.7%)	0.123
Unhealthy	42 (25%)	53 (32.3%)	
Types of unhealthy weight-control behaviors			
Vomit	7 (16.7%)	13 (25%)	0.042^*∗*^
Fasted for long hours	25 (59.5%)	28 (52.5%)	
Took diet pills		3 (5.6%)	
Used food substitute	10 (23.8%)	9 (16.9%)	

^#^Chi-square test, $*t*-test,  ^*∗∗*^*P* ≤ 0.001, and ^*∗*^*P* ≤ 0.05.

**Table 3:** The calorie intake and weight concern according to body weight perception among the groups (*N* = 333).

Body weight perception	Calories	*P*	Weight concern	*P*

Control group, 169 (50.8%)				
Underperception, 29 (17.2%)	636.65 ± 244.77		5.37 + 2.54	
Accurate perception, 105 (62.1%)	566.79 ± 228.06	0.331	6.42 ± 2.87	0.009^*∗*^
Overperception, 35(20.7%)	599.39 ± 239.15		8.71 ± 2.76	
Experimental group, 164 (49.2%)				
Underperception, 13 (7.9%)	513.84 ± 178.58	0.029^*∗*^	6.38 ± 3.25	0.036^*∗*^
Accurate perception, 82 (50%)	567.56 ± 203.36		7.91 ± 3.22	
Overperception, 69 (42.1%)	479.15 ± 203.79		9.27 ± 2.95	

^*∗*^*P* ≤ 0.05.

## Data Availability

The datasets used and analyzed during the present study are available from the corresponding author on reasonable request.
